# Structures of the xyloglucans in the monocotyledon family Araceae (aroids)

**DOI:** 10.1007/s00425-023-04071-w

**Published:** 2023-01-17

**Authors:** Shih-Yi Hsiung, Jing Li, Balazs Imre, Mu-Rong Kao, Hsien-Chun Liao, Damao Wang, Chih-Hui Chen, Pi-Hui Liang, Philip J. Harris, Yves S. Y. Hsieh

**Affiliations:** 1 Division of Glycoscience, Department of Chemistry, School of Engineering Sciences in Chemistry, Biotechnology and Health, KTH Royal Institute of Technology, AlbaNova University Centre, 106 91 Stockholm, Sweden; 2 School of Pharmacy, College of Pharmacy, Taipei Medical University, Taipei, Taiwan; 3 College of Life Science, Shanghai Normal University, Shanghai, China; 4 Division of Botany, Taiwan Endemic Species Research Institute, Nantou, 552 Taiwan; 5 College of Food Science, Southwest University, Chongqing, China; 6 School of Pharmacy, College of Medicine, National Taiwan University, Taipei, Taiwan; 7 School of Biological Sciences, The University of Auckland, Private Bag 92019, Auckland, 1142 New Zealand

**Keywords:** Duckweeds, Fucosylation, Mass spectrometry, Plant cell walls, Xyloglucanase, Xyloglucan oligosaccharides

## Abstract

**Main conclusion:**

The xyloglucans of all aquatic Araceae species examined had unusual structures compared with those of other non-commelinid monocotyledon families previously examined.

**Abstract:**

The aquatic Araceae species *Lemna minor* was earlier shown to have xyloglucans with a different structure from the fucogalactoxyloglucans of other non-commelinid monocotyledons. We investigated 26 Araceae species (including *L. minor*), from five of the seven subfamilies. All seven aquatic species examined had xyloglucans that were unusual in having one or two of three features: < 77% XXXG core motif [*L. minor* (Lemnoideae) and *Orontium aquaticum* (Orontioideae)]; no fucosylation [*L. minor* (Lemnoideae), *Cryptocoryne aponogetonifolia*, and *Lagenandra ovata* (Aroideae, Rheophytes clade)]; and > 14% oligosaccharide units with S or D side chains [*Spirodela polyrhiza* and *Landoltia punctata* (Lemnoideae) and *Pistia stratiotes* (Aroideae, *Dracunculus* clade)]. Orontioideae and Lemnoideae are the two most basal subfamilies, with all species being aquatic, and Aroideae is the most derived. Two terrestrial species [*Dieffenbachia seguine* and *Spathicarpa hastifolia* (Aroideae, *Zantedeschia* clade)] also had xyloglucans without fucose indicating this feature was not unique to aquatic species.

**Supplementary Information:**

The online version contains supplementary material available at 10.1007/s00425-023-04071-w.

## Introduction

Xyloglucans (XGs) are a family of non-cellulosic polysaccharides (hemicelluloses) that occur in the primary cell walls of all land plants (embryophytes) and in the most recently diverged lineages of their ancestors, the charophycean green algae (Fry 1989, 2010; Scheller and Ulvskov 2010; Pauly and Keegstra 2016; Brennan et al. 2019; Mikkelsen et al. 2021). However, the proportion of XG in primary cell walls of vegetative organs varies with phylogenetic position (Hsieh and Harris 2009; Pauly and Keegstra 2016). For example, in angiosperms, the proportion of XG varies from a high (~ 20–25% dry mass) in most eudicotyledons to only a small proportion (~ 2–5% dry mass) in the monocotyledon family Poaceae (grasses including cereals) (Fry 1989).

**Fig. 1:**
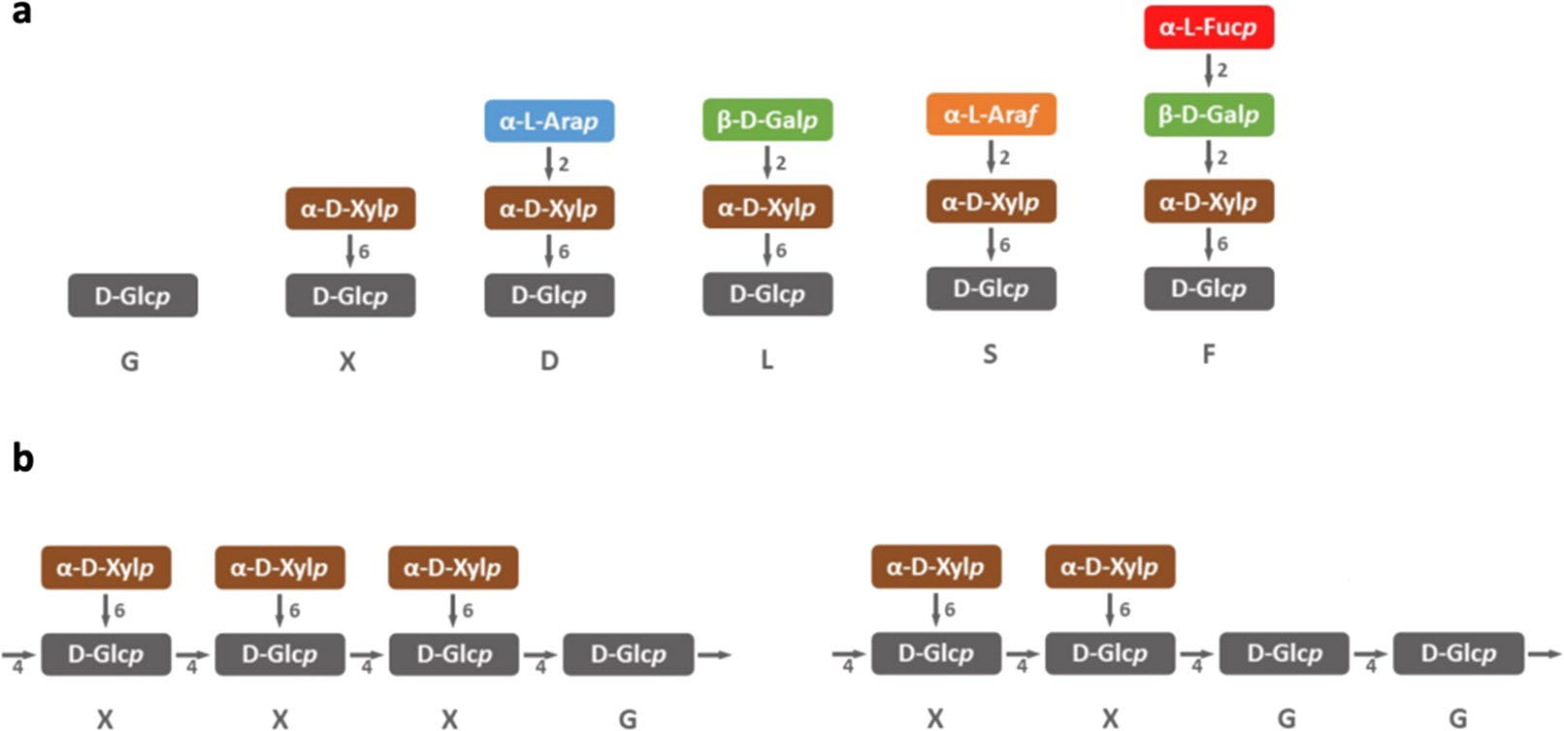
**a** Single-letter codes to describe xyloglucan side chains mentioned in the text. **b** Structures of repeating XXXG and XXG core motifs; the xylose residues may have other glycosyl residues attached

Structurally, XGs consist of a backbone of (1 → 4)-linked β-d-glucopyranose (β-d-Glc*p*) residues with some of these having an α-xylopyranose (α-Xyl*p*) residue attached to them at *O*-6. Other substituents may be present on some of the α-Xyl*p* residues, giving a number of different side chains on the glucan backbone. A single-letter code is used to describe the structures of these side chains (Fry et al. 1993; Schultink et al. 2014; Tuomivaara et al. 2015), with G being used to describe an unsubstituted Glc residue in the backbone and X to describe a Glc residue substituted with an α-Xyl*p* residue attached at O-6 (Fig. 1a). A total of over 20 xyloglucan side chains have been described (Schultink et al. 2014; Tuomivaara et al. 2015), with the most frequent side chains in angiosperm xyloglucans being X, L and F (Fig. 1a).

A commonly used approach to structurally analyse XGs is to treat them with an XG-specific *endo*-(1 → 4)-β-glucanase (XEG), or less specifically with an *endo*-cellulase [*endo*-(1 → 4)-β-glucanase] and to identify and quantify the oligosaccharides released (Vincken et al. 1997). These enzymes usually cleave the backbone between G and X, and from the XGs in the primary cell walls of vegetative organs of most eudicotyledons release a mixture of three major oligosaccharides XXXG, XXFG and XLFG, with smaller amounts of XXLG, XLXG and XLLG (Harris 2005). Such XGs have a repeating XXXG core motif, contain galactose and fucose, and are referred to as fucogalactoxyloglucans. The proportions of the different oligosaccharide components vary somewhat with the vegetative organ examined within a particular plant (Pauly et al. 2001). Similar XGs, but without fucose, known as galactoxyloglucans, occur in high proportions in the thick cell walls of cotyledons in the seeds of many eudicotyledons (Harris 2005; Gidley and Reid 2006; Nishinari et al. 2007; Buckeridge 2010). A commercially available galactoxyloglucan is obtained from the seeds of tamarind (*Tamarindus indica*; family Fabaceae) (Nishinari et al. 2007).

XGs with a different repeating core motif, XXGG, occur in the eudicotyledon tobacco and potato family Solanaceae (order Solanales) (Harris 2005; Hoffman et al. 2005; Hsieh and Harris 2009; Lampugnani et al. 2013). These XGs have S, but not F side chains (Fig. 1a) and oligosaccharides obtained from potato (*Solanum tuberosum*) XG included XXGG, XSGG, XLGG and LSGG (Vincken et al. 1996, 1997). The same repeating core motif also occurs in the monocotyledon family Poaceae (grasses including cereals), but in addition to XXGG, the core motifs XXG, XXGGG and XXGGGG occur together with small amounts of XXGGGGG (Fry 1989; Gibeaut et al. 2005; Hsieh and Harris 2009). We refer to the repeating core motif as XXG_n_, where *n* = ~ 1–5 (Hsieh and Harris 2009). XG oligosaccharides (XGOs) with this repeating core motif obtained from Poaceae cell walls may also contain L side chains, e.g. XLGG (Hsieh and Harris 2009). However, the XGs of some Poaceae species and specific cell types yield some XGOs with the XXXG core motif (Hsieh and Harris 2009; Liu et al. 2015).

Initially, research on the structures of XGs in monocotyledons focused mostly on grasses (Poaceae). However, in contrast to Poaceae XG, it was also established that the XGs of *Allium cepa* (onion), *A. sativa* (garlic) and their hybrid (family Amaryllidaceae, order Asparagales) are fucogalactoxyloglucans similar to those in the primary cell walls of most eudicotyledons (Ohsumi and Hayashi 1994). More recently, a study (Hsieh and Harris 2009) examined the structures of the XGs in the primary vegetative cell walls of a range of monocotyledon species selected on their phylogenetic positions.

**Fig. 2:**
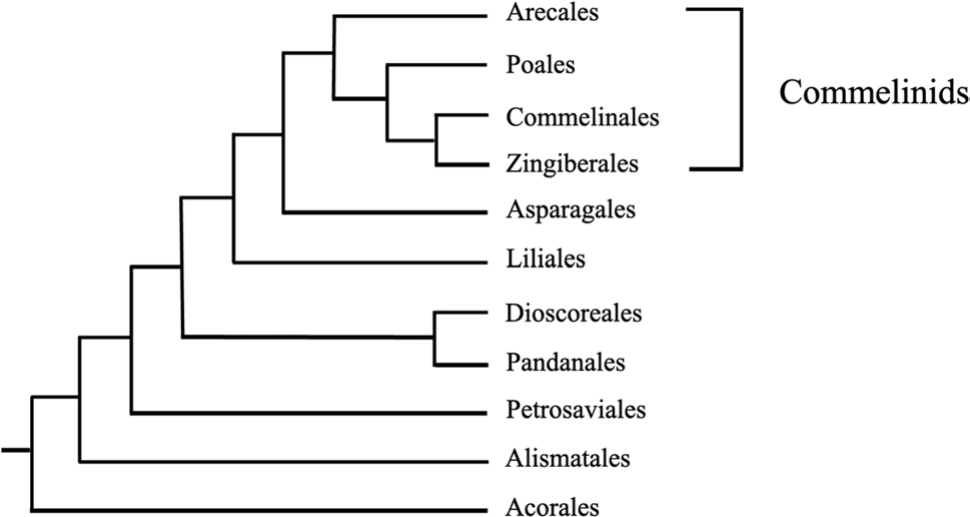
Phylogeny of monocotyledon orders based on APG IV (2016)

Phylogenetic studies of monocotyledons using the nucleotide sequences of genes have shown four groups in phylogenetic sequence from basal to most derived: the Acorales, the Alismatales, the liliids or petaloid monocotyledons and the commelinid monocotyledons (Chase et al. 2006; Hsieh and Harris 2009; AGP IV 2016) (Fig. 2). Acorales is the basal order in the monocotyledons and is composed of only one family and one genus *Acorus*, which comprises two emergent aquatic species. The Alismatales is a sister to the rest of the monocotyledons and consists of the large family Araceae and 13 smaller families. The liliids are composed of five orders in phylogenetic sequence: Petrosaviales, Pandanales (screw pines, etc.), Dioscoreales (yams), Liliales (lilies, etc.), and Asparagales (onion, asparagus, etc.). The Asparagales is a sister to the fourth group, the commelinid monocotyledons, first recognized chemically and histochemically by the presence of ester-linked ferulic acid in their primary cell walls (Harris and Hartley 1980; Harris 2005; Harris and Trethewey 2010). This group is composed of the basal order Arecales [Arecaceae (the palms) and Dasypogonaceae] and the Poales (grasses, rushes, sedges, bromeliads, etc.), which is a sister to the Commelinales (spiderworts, etc.) and Zingiberales (gingers, etc.).

In their study of monocotyledon XGs, Hsieh and Harris (2009) treated an alkali extract of cell walls [as alcohol-insoluble residues (AIRs)] with an XEG to release XGOs. All types of XGs so far examined, except those from the thick cotyledon cell walls of seeds, have *O*-acetyl groups on specific glycosyl residues (Hsieh and Harris 2009; Gille and Pauly 2012). Using an alkali extract results in the removal of these *O*-acetyl groups from the XGs, so simplifying the analysis. The XGOs were analysed semi-quantitatively using both high-performance anion-exchange chromatography (HPAEC) with pulsed amperometric detection (PAD) and matrix-assisted laser-desorption ionization time-of-flight mass spectrometry (MALDI-TOF MS) (Lerouxel et al. 2002; Günl et al. 2010; Tuomivaara et al. 2015). Both methods gave similar results and showed that the most diverse XG structures were within the commelinid monocotyledons. Within the Poales, the Poaceae had mostly XGs with a repeating XXG_*n*_ core motif and no F side chains, although a few species had small proportions of the XXXG core motif. Some of the other Poales families had XGs with both XXXG and XXG_*n*_ core motifs, and others only XXXG; XXFG oligosaccharides, but no XLFG were released. The Commelinales and Zingiberales also had XGs with both XXXG and XXG_n_ core motifs, with small proportions of XXFG oligosaccharides, but no XLFG was released. However, the XGs in the basal order Arecales had only the XXXG core motif with both XXFG and XLFG oligosaccharides, similar to the eudicotyledon fucogalactoxyloglucans. For the orders of non-commelinid monocotyledons examined, the Asparagales had similar XGs to the Arecales except for *Vanilla pompona* (Orchidaceae), which had some XXG_n_ core motif (23%) (the percentages cited are from the MALDI-TOF MS analyses) although mostly XXXG core motif (77%) and a much smaller proportion (6%) of the fucosylated oligosaccharides (XXFG and XLFG). The species examined in the Liliales, Dioscoreales, and Pandanales also had XGs with mostly the XXXG core motif, but with some of the XXG_n_ core motif (11%, 22%, and 10%, respectively), with the fucosylated oligosaccharides XXFG and XLFG being released.

No representative of the order Petrosaviales was examined, but two species were examined in the Alismatales both in the aroid family Araceae: the terrestrial species *Zantedeschia aethiopica* and the aquatic species *Lemna minor. Z. aethiopica* XG had a similar XGO profile to eudicotyledon fucogalactoxyloglucans with only the XXXG core motif, although the total percentage for the two fucosylated XGOs was quite low at 11%. However, the XGO profile of the floating aquatic duckweed species *Lemna minor* was quite different, with a high proportion of the XXG_n_ core motif (80%) and neither of the two fucosylated XGOs, XXFG and XLFG.

**Fig. 3:**
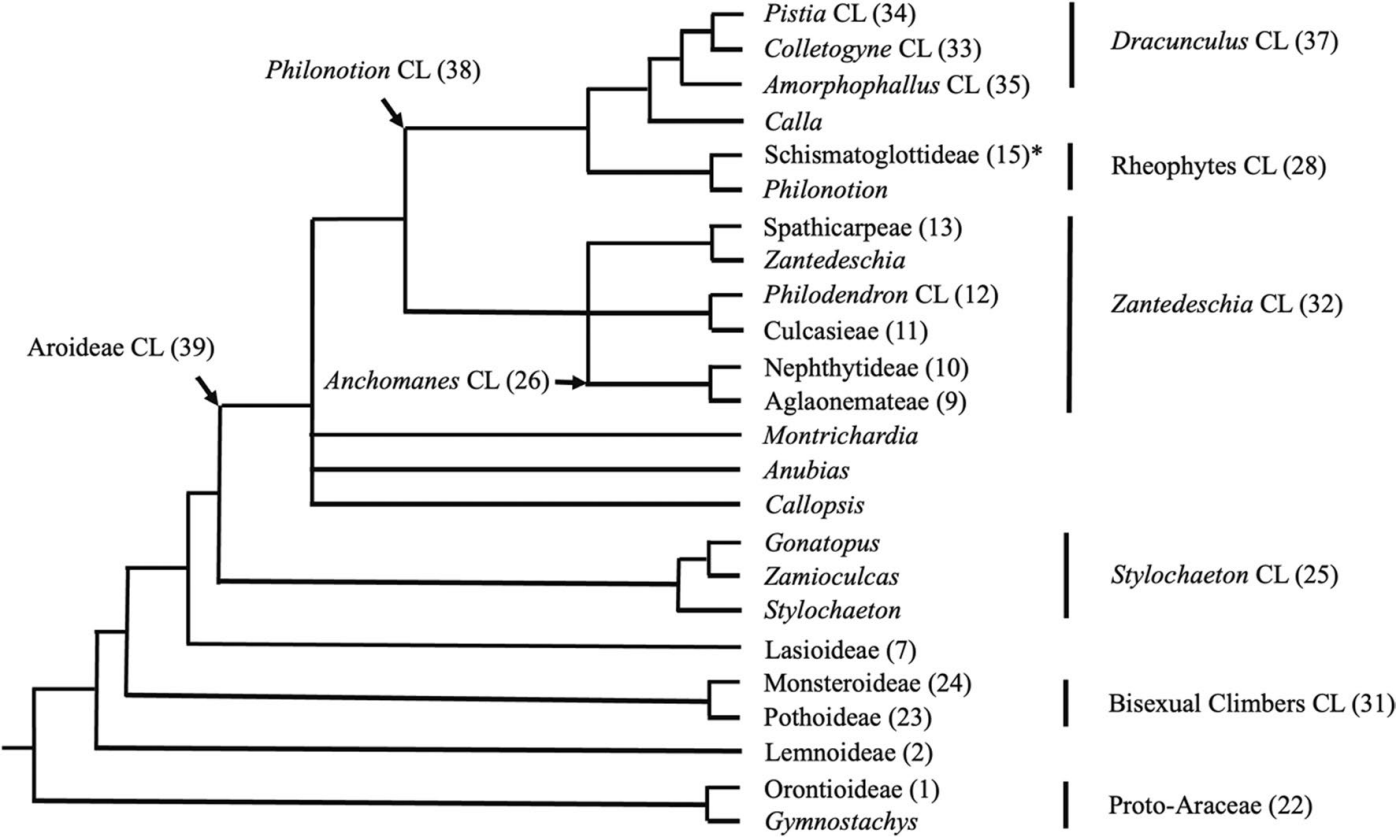
Simplified molecular phylogeny of family Araceae from Cusimano et al. (2011) showing major clades (CL) (with numbers) based on Mayo et al. (2013). The clade name may be a subfamily (ending in oideae), a tribe (ending in eae), or an informal name, with the circumscription of each clade described in Table 1 of Cusimano et al. (2011). The genera *Gonatopus* and *Zamioculcas* comprise subfamily Zamioculcadoideae. *Cryptocoryneae (CL 14) is not shown; it is a small sister clade to Schismatoglottideae (CL 15) and comprises the genera *Cryptocoryne* and *Lagenandra*

Because of these quite different XG structures for the two species examined in the Araceae, we have examined the structures of the XGs in other Araceae species, with sampling based on Araceae phylogeny. The Araceae is a large, mostly tropical family of herbaceous plants, with ~ 114 genera and ~ 3750 species (Christenhusz and Byng 2016) and a range of habits and life forms that are mostly terrestrial but some aquatic (Croat 1988; Mayo et al. 1998; Cabrera et al. 2008). The molecular phylogeny of Araceae has been investigated most recently by Cabrera et al. (2008), Cusimano et al. (2011), and Henriquez et al. (2014). Cabrera et al. (2008) carried out a phylogenetic analysis on almost all Araceae genera using five regions of plastid DNA. Importantly, this was the first study to show that the duckweeds (*Lemna* and four other genera) formerly regarded as a separate family, Lemnaceae, are part of the Araceae. Cusimano et al. (2011) used the data set of Cabrera et al. (2008), but this was augmented by further sequences. In their phylogeny, they identified 44 robust, numbered molecular clades, which were also supported by morphological data. A simplified version of this phylogeny is shown in Fig. 3. Whole chloroplast sequencing (Henriquez et al. 2014) gave the same overall phylogeny although there were some differences in detail particularly within the Aroideae. Seven subfamilies were recognized by Cusimano et al. (2011): Orotioideae, Lemnoideae, Pothoideae, Monsteroideae, Lasioideae, Zamioculcadoideae and Aroideae. The subfamily Aroideae is by far the largest with ~ 75 genera and ~ 2580 species (Stevens 2001).

The two species for which the XGs had previously been examined *L. minor* and *Z. aethiopica* are in the subfamilies Lemnoideae and Aroideae, respectively. The early diverging subfamily Lemnoideae and basal subfamily Orotioideae (Fig. 3) have much simpler morphologies than later diverging subfamilies and both subfamilies are aquatic, whereas Z. *aethiopica* is a terrestrial species (Cabrera et al. 2008). However, the aquatic habit also occurs as a secondary acquisition in the Lasioideae and some Aroideae species including water lettuce *Pistia stratiotes* in the *Dracunculus* clade (*Pistia* subclade) and species in the Rheophytes clade (Fig. 3) (Cabrera et al. 2008). It is thus possible that if the unusual structural features of *L. minor* XG result from an adaptation to the aquatic environment, these features may also be present in taxa where the aquatic habit occurs as an independent secondary acquisition.

In the present study, we examined the structures of the XGs of 26 species from all subfamilies of Araceae except Lasioideae and Zamioculcadoideae to determine if they had high proportions of the repeating XXG_n_ core motif and no F side chains. In particular, we examined the XGs of the duckweeds *Landoltia punctata*, *Spirodela polyrhiza* (in addition to *L. minor*) in the aquatic, early diverging subfamily Lemnoideae and *Orontium aquaticum* in the aquatic, basal subfamily Orotioideae (Fig. 3). We also examined the XGs of three aquatic species in the subfamily Aroideae: *P. stratiotes* (*Dracunculus* clade) and *Cryptocoryne aponogetonifolia* and *Lagenandra ovata* (Rheophytes clade) (Fig. 3). The other species examined were neither aquatic nor members of the two earliest emerging subfamilies Orotioideae or Lemnoideae. The hypothesis was that the XGs of all aquatic Araceae species have low proportions (< 77%) of the repeating XXXG core motif [high proportions (> 23%) of the repeating XXG_n_ core motif] and no F side chains. We analysed the XGs using the methods of Hsieh and Harris (2009) except an XEG preparation from *Paenibacillus* sp. (Yaoi et al. 2005; Steck et al. 2021) was used instead of from *Aspergillus aculeatus* (Pauly et al. 1999) and only MALDI-TOF MS was used in a semi-quantitative way to profile the XGOs released (Lerouxel et al. 2002; Hsieh and Harris 2009, 2012; Tuomivaara et al. 2015).

## Materials and methods

### Plant materials

**Fig. 4:**
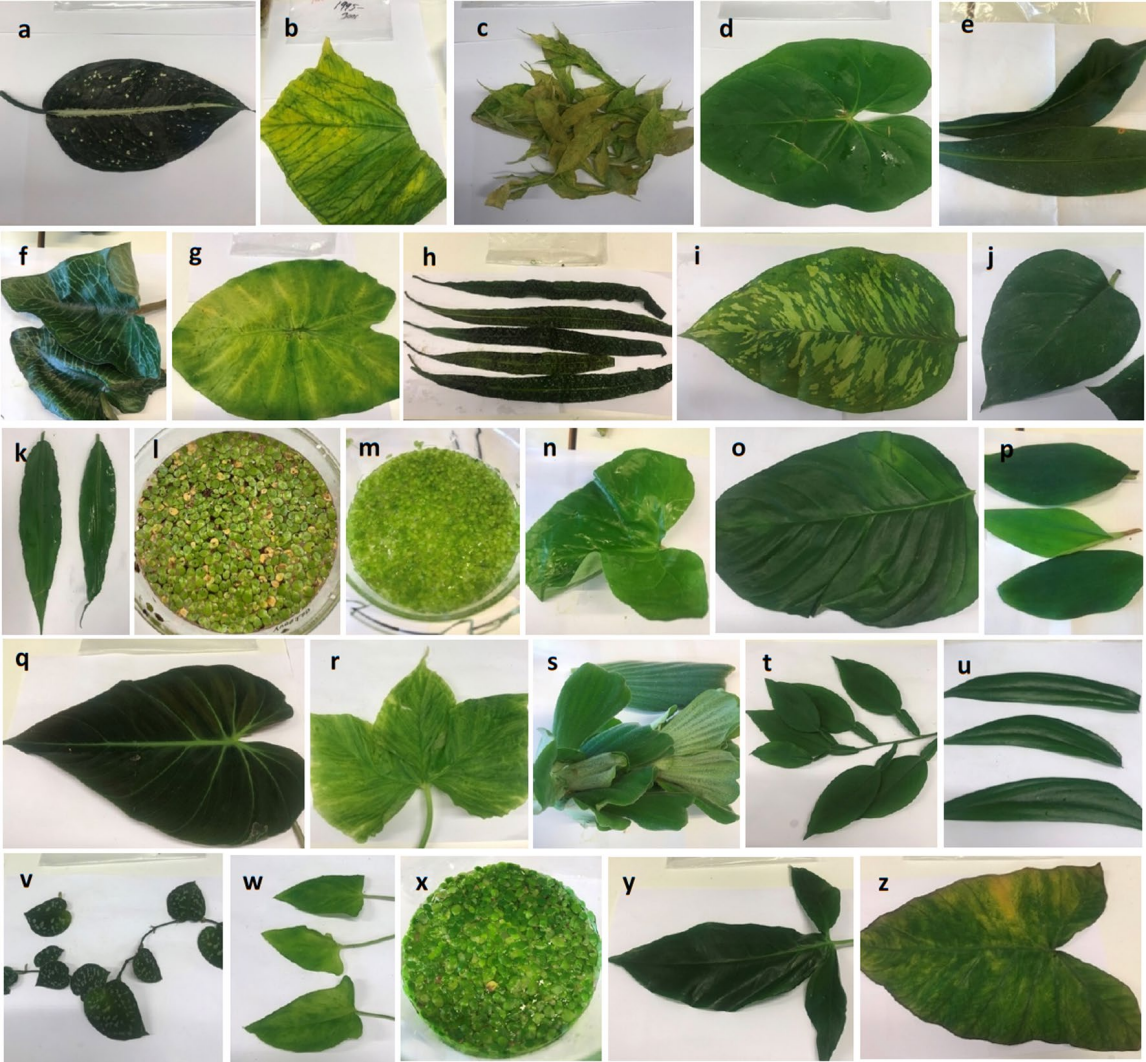
Photographs of plant materials used to prepare AIRs from all species examined (genera arranged in alphabetical order). **a** *Aglaonema costatum*. **b** *Alocasia macrorrhizos.* **c** *Amorphophallus dunnii*. **d** *Anthurium andraeanum*. **e** *Anthurium gracile*. **f** *Arum pictum.* **g** *Colocasia esculenta*. **h** *Cryptocoryne aponogetonifolia*. **i** *Dieffenbachia seguine*. **j** *Epipremnum aureum*. **k** *Lagenandra ovate*. **l** *Landoltia punctate*. **m** *Lemna minor*. **n** *Leucocasia gigantean*. **o** *Monstera adansonii*. **p** *Orontium aquaticum*. **q** *Philodendron hederaceum*. **r** *Pinellia tripartite.* **s** *Pistia stratiotes*. **t** *Pothos scandens*. **u** *Rhaphidophora decursiva*. **v** *Scindapsus pictus*. **w** *Spathicarpa hastifolia*. **x** *Spirodela polyrhiza*. **y** *Syngonium auritum*. **z** *Xanthosoma sagittifolium*

In the present study, we examined 26 species of Araceae. The species, clade numbers and names, as used by Cusimano et al. (2011), the sources of the species, the organs that were used to isolate AIRs and the habits/life forms of the species are shown in Table 1. The organs used were all leaves, with the thalli of the duckweed species (subfamily Lemnoideae) often being considered as modified leaves. Photographs of the plant materials are shown in Fig. 4. Leaves (5–20) were collected from each of 5–10 plants of each species (for the duckweeds at least 50 thalli of each species) and pooled to isolate AIRs for analysis. This was done to reduce the effects of any variation in xyloglucan structure in any individual leaf or in the leaves on any individual plant.

**Table 1:** Species, clade numbers and names, sources, organs used, and habits and life forms

Species	Clade no. and name^a^	Sources^b^	Organs	Habits and life forms^c^
*Orontium aquaticum* L.	22, Proto-Araceae; 1, Orontioideae	SE	Leaf blades	Partly submerged aquatic (emergent)
*Spirodela polyrhiza* (L.) Schleid.	22, Proto-Araceae; 2, Lemnoideae	SE	Thalli^d^	Free floating aquatic
*Landoltia punctata* (G. May) Les & D.J. Crawford	22, Proto-Araceae; 2, Lemnoideae	AK^b^	Thalli^d^	Free floating aquatic
*Lemna minor* L	22, Proto-Araceae; 2, Lemnoideae	SE	Thalli^d^	Free floating aquatic
*Pothos scandens * L.	31, Bisexual Climbers CL; 23, Pothoideae; 3, Potheae	UU	Leaf blades	Climbing hemiepiphyte
*Anthurium andraeanum* Linden ex André	31, Bisexual Climbers CL; 23, Pothoideae	UU	Leaf blades^e^	Climbing hemiepiphyte
*Anthurium gracile* (Rudge) Lindl.	31, Bisexual Climbers CL; 23, Pothoideae	SE	Leaf blades	Climbing hemiepiphyte
*Rhaphidophora decursiva* (Roxb.) Schott	31, Bisexual Climbers CL; 24, Monsteroideae; 6, *Rhaphidophora* CL	UU	Leaf blades^e^	Climbing hemiepiphyte
*Scindapsus pictus* Hassk.	31, Bisexual Climbers CL; 24, Monsteroideae; 6, *Rhaphidophora* CL	UU	Leaf blades	Climbing hemiepiphyte
*Epipremnum aureum* (Linden & André) G.S. Bunting	31, Bisexual Climbers CL; 24, Monsteroideae; 6, *Rhaphidophora* CL	UU	Leaf blades^e^	Climbing hemiepiphyte
*Monstera adansonii* Schott	31, Bisexual Climbers CL; 24, Monsteroideae; 6, *Rhaphidophora* CL	UU	Leaf blades	Climbing hemiepiphyte
*Aglaonema costatum* N.E. Br.	39, Aroideae CL; 32, *Zantedeschia* CL; 26, *Anchomanes* CL; 9, Aglaonemateae	UU	Leaf blades^e^	Terrestrial caulescent herb
*Philodendron hederaceum* (Jacq.) Schott	39, Aroideae CL; 32, *Zantedeschia* CL; 12, *Philodendron* CL	UU	Leaf blades^e^	Climbing hemiepiphyte
*Dieffenbachia seguine* (Jacq.) Schott	39, Aroideae CL; 32, *Zantedeschia* CL; 13, Spathicarpeae	UU	Leaf blades^e^	Terrestrial caulescent herb
*Spathicarpa hastifolia* Hook.	39, Aroideae CL; 32, *Zantedeschia* CL; 13, Spathicarpeae	UU	Leaves	Terrestrial tuberous geophyte
*Cryptocoryne aponogetonifolia* Merr.	39, Aroideae CL; 38, *Philonotion* CL; 28, Rheophytes CL; 14, Cryptocoryneae	UU	Leaf blades^e^	Submerged aquatic
*Lagenandra ovata* (L.) Thwaites	39, Aroideae CL; 38, *Philonotion* CL; 28, Rheophytes CL; 14, Cryptocoryneae	UU	Leaf blades^e^	Submerged aquatic or marsh plant (helophyte)
*Amorphophallus dunnii* Tutcher	39, Aroideae CL; 38, *Philonotion* CL; 37, *Dracunculus* CL; 35, *Amorphophallus* CL; 16, Thomsonieae	UU	Leaf blades	Terrestrial tuberous geophyte
*Syngonium auritum* (L.) Schott	39, Aroideae CL; 38, *Philonotion* CL; 37, *Dracunculus* CL; 35, *Amorphophallus* CL; 17, Caldieae	UU	Leaf blades^e^	Climbing hemiepiphyte
*Xanthosoma sagittifolium* (L.) Schott	39, Aroideae CL; 38, *Philonotion* CL; 37, *Dracunculus* CL; 35, *Amorphophallus* CL; 17, Caldieae	UU	Leaf blades^e^	Terrestrial tuberous geophyte
*Pistia stratiotes* L.	39, Aroideae CL; 38, *Philonotion* CL; 37, *Dracunculus* CL; 34, *Pistia* CL	UU	Whole plant^d^	Free-floating aquatic
*Colocasia esculenta* (L.) Schott	39, Aroideae CL; 38, *Philonotion* CL; 37, *Dracunculus* CL; 34, *Pistia* CL; 20, *Colocasia* CL	UU	Leaf blades^e^	Terrestrial tuberous geophyte
*Leucocasia gigantea* (Blume) Schott	39, Aroideae CL; 38, *Philonotion* CL; 37, *Dracunculus* CL; 34, *Pistia* CL; 20, *Colocasia* CL	SE	Leaf blades (young)	Terrestrial tuberous geophyte
*Alocasia macrorrhizos* (L.) G.Don	39, Aroideae CL; 38, *Philonotion* CL; 37, *Dracunculus* CL; 34, *Pistia* CL; 30, *Alocasia* CL	UU	Leaf blades	Terrestrial rhizomatous geophyte
*Pinellia tripartita* (Blume) Schott	39, Aroideae CL; 38, *Philonotion* CL; 37, *Dracunculus* CL; 34, *Pistia* CL; 30, *Alocasia* CL	UU	Leaf blades	Terrestrial tuberous geophyte
*Arum pictum* L.f	39, Aroideae CL; 38, *Philonotion* CL; 37, *Dracunculus* CL; 34, *Pistia* CL; 30, *Alocasia* CL; 21, Areae	SE	Leaf blades^e^	Terrestrial tuberous geophyte

^a^Clade numbers and names are from Cusimano et al. (2011) and Mayo et al. (2013) and are also used in Fig. 3

^b^Sources of the plants were as follows: UU = Uppsala linneanska trädgårdar, Uppsala Universitet, Uppsala, Sweden, and collected by Jesper Kårehed; SE = Bergianska trädgården, Stockholm Universitet, Stockholm, Sweden, and collected by Gunvor Larsson; AK = Collected in Auckland region of New Zealand

^c^Habits and life forms of the plants are mostly from Croat (1988)

^d^Roots removed

^e^Major vascular bundles with lignified cell walls removed

### Isolation of AIRs

AIRs were isolated following the procedure of Popper et al. (2001) as modified by Hsieh and Harris (2009, 2012). Whole leaves were used if they contained mostly non-lignified cell walls, or leaves were used from which tissues containing cells with lignified walls had been removed by cutting (Table 1). Lignified cell walls were detected histochemically using bright-field light microscopy of fresh, transverse sections (cut by hand with a razor blade) by the red colour reaction given by phloroglucinol–HCl (the Wiesner colour reagent) (Harris et al. 1980). The plant material (1–20 g fresh weight) was homogenized in 70% aqueous ethanol (50 ml at 4 °C) using a homogenizer (Model SH-HZA, SH Scientific, Sejong, South Korea). The homogenate was filtered onto Miracloth (Calbiochem, Darmstadt, Germany), then ground into a fine powder in liquid nitrogen using a ceramic mortar and pestle (both pre-cooled to -80 °C). The powder was incubated in 70% ethanol (150 ml at 60 °C) for 6 h to remove soluble sugars. The suspension was filtered onto fresh Miracloth, and the residue washed with 70% ethanol (100 ml) followed by acetone (100 ml), dried in a stream of air, and stored over silica gel.

### Extraction of non-cellulosic polysaccharides from AIRs

This was carried out as described by Hsieh and Harris (2009, 2012). The AIRs (70–100 mg) were extracted, on an orbital shaker at 37 °C for 16 h, with 6 M NaOH containing 1% NaBH_4_ (10 ml) to prevent possible β-oxidation of the polysaccharides and alkaline peeling. The suspension was then filtered using Miracloth, and the filtrate cooled on ice for 20 min before it was adjusted to pH 5.0 by adding 17.4 M acetic acid. One drop of toluene was added to the solution to stop microbial growth, before it was dialysed, first against tap water at room temperature for 24 h, and then against distilled water for 1 h. The resulting dialysate was freeze dried.

### Treatment of extracted non-cellulosic polysaccharides with XEGs

The extracted non-cellulosic polysaccharide preparations (1–2 mg) were incubated with a high purity, recombinant XEG (GH5) (EC 3.2.1.151) from *Paenibacillus* sp. (Megazyme International Ireland Ltd, County Wicklow, Ireland) (10U) in 100 mM sodium acetate buffer (pH 5.5) at 40 °C for 20 min. The samples were then heated to 100 °C for 2 min to stop the reaction. Buffer-only control incubations were also carried out.

The sample (2 mg) of extracted non-cellulosic polysaccharides from the AIR of *L. punctata* was an exception in that it was treated with recombinant XEG from *A. aculeatus* (EC 3.2.1.151) exactly as described by Hsieh and Harris (2009, 2012).

### MALDI-TOF MS

A MALDI-TOF mass spectrometer (Model BioTOF Ultraflex II, Bruker Daltonics, Billeriaca, MA, USA) was used to identify the various XGOs by determining the molecular weights of the sodium adduct ions [M + Na]^+^ of the oligosaccharides. An exception was the sample from *L. punctata* for which the spectrometer described in Hsieh and Harris (2009, 2012) was used. XEG-treated samples (10 µl) were mixed with 2,5-dihydroxybenzoic acid (10 µl, 10 mg ml^−1^ in water) and NaCl (6 µl, 10 mM in water), and an aliquot (1 µl) was dried onto a sample plate. A reference preparation of tamarind XGOs (Megazyme) (10 µl) (a mixture of XXXG, XLXG, XXLG and XLLG 3.67 mg ml^−1^ in water) was treated in the same way. The spectrometer was operated in the reflectron mode at an accelerating voltage of 20 kV with a delay time of 200 ns. For each spectrum, data from 1000–2000 laser shots were averaged using Bruker flexAnalysis software. A 6 M NaOH extract of an AIR of hypocotyls of the eudicotyledon *Vigna radiata* (family Fabaceae) was also treated with the XEG from *Paenibacillus* sp. as above and, after mass spectrometry, sodium adduct ions [M + Na]^+^ of the oligosaccharides were compared with those obtained from a similar extract treated with the XEG from *A. aculeatus* in the study of Hsieh and Harris (2009). The XGOs XXSG/XXDG and XLSG/XLDG containing the S or D side chain, were identified by comparison with ions obtained in the study of Hsieh and Harris (2012). A semi-quantitative measurement was made of the proportion of each XGO by expressing the intensity of its [M + Na]^+^adduct ion (on a height basis) as a percentage of the total intensities of ions identified as [M + Na]^+^ adduct ions of XGOs; absolute response factors were not determined.

## Results

The profile of XGOs released by the XEG preparation from *Paenibacillus* sp. from the 6 M NaOH extract of the AIR of the reference eudicotyledon *V. radiata* had high proportions of sodium adduct ions [M + Na]^+^ corresponding to XXXG, XXFG and XLFG (XXG 2%, XXXG 41%, XLXG/XXLG 5%, XXFG 41%, XLLG trace and XLFG 10%) and was similar to the profile released by the XEG preparation from *A. aculeatus* from a similar extract from the same species (Hsieh and Harris 2009).

**Fig. 5:**
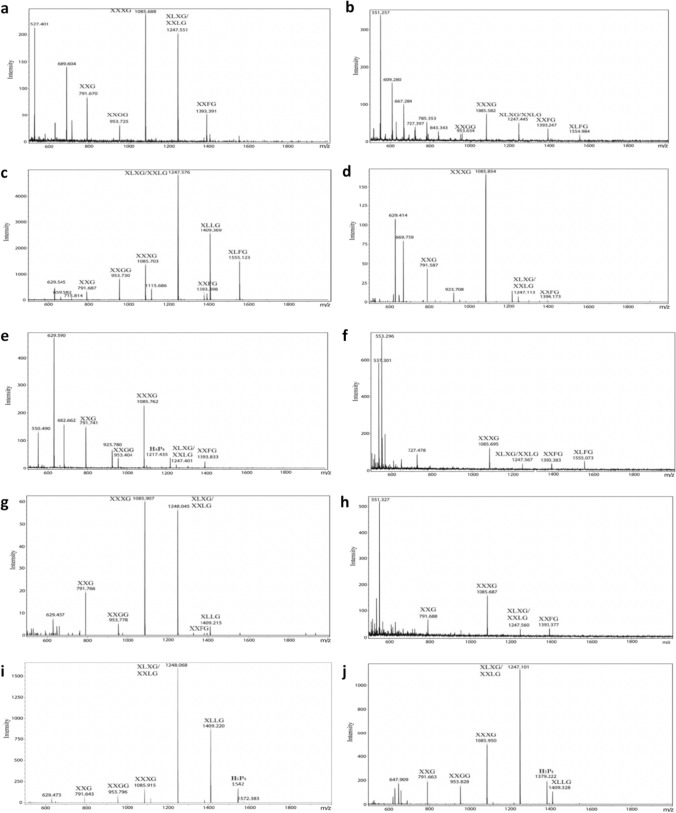
MALDI-TOF mass spectra of xyloglucan oligosaccharides obtained from AIRs from ten of the species examined, treated with XEG (genera arranged in alphabetical order); HxPy = hexose x and pentose y). **a** *Aglaonema costatum* (subfamily Aroideae). **b** *Alocasia macrorrhizos* (subfamily Aroideae). **c** *Amorphophallus dunnii* (subfamily Aroideae). **d** *Anthurium andraeanum* (subfamily Pothoideae). **e** *Anthurium gracile* (subfamily Pothoideae). **f** *Arum pictum* (subfamily Aroideae). **g** *Colocasia esculenta* (subfamily Aroideae). **h** *Leucocasia gigantea* (subfamily Aroideae). **i** *Cryptocoryne aponogetonifolia* (subfamily Aroideae). **j** *Dieffenbachia seguine* (subfamily Aroideae)

The relative proportions of XGOs released from the AIR extracts of the Araceae species examined determined by MALDI-TOF MS are shown in Table 2, with the mass spectra shown in Fig. 5 and Fig. S1. The repeating XXXG core motif accounted for 23–100% of the XGO profiles but, except for three species (*Orontium aquaticum*, *L. minor* and *Anthurium gracile*), accounted for > 76%. Most profiles had the XXXG in high proportions, XLXG/XXLG in varying proportions, and the fucosylated XXFG. Only a few profiles in subfamily Monsteroideae and subfamily Aroideae, *Dracunculus* clade also had XLFG, and the profiles of five species (*L. minor*, *Dieffenbachia seguine*, *Spathicarpa hastifolia*, *Cryptocoryne aponogetonifolia* and *Lagenandra ovata*) had neither XLFG nor XXFG. The profiles of some species had XLLG, but in varying proportions. Except for one species *Arum pictum* (subfamily Aroideae, *Dracunculus* clade), the XGs of all had some repeating XXG_n_ core motif, with most of the XGOs with this core motif being XXG and XXGG. Although XXG oligosaccharides are likely to be released directly from the XGs, it is possible they result from further action of the XEG preparation on XXGG as it has been found that the *Paenibacillus* sp. XEG preparation can degrade XSGG into XSG (Steck et al. 2021).

**Table 2:** Proportions of xyloglucan oligosaccharides in xyloglucan-specific *endo*-(1 → 4)-β-glucanase hydrolysates as determined by MALDI-TOF MS

Taxon^a^					Proportions of xyloglucan oligosaccharides^b^									
	791 H_3_P_2_ XXG	953 H_4_P_2_ XXGG	1115 H_5_P_2_ XXGGG/ XLGG	1085 H_4_P_3_ XXXG	1217 H_4_P_4_ XXSG/ XXDG	1247 H_5_P_3_ XLXG/XXLG	1379 H_5_P_4_ XLSG/ XLDG	1393 H_5_P_3_ dH_1_ XXFG	1409 H_6_P_3_ XLLG	1555 H_6_P_3_ dH_1_ XLFG	XXFG + XLFG	XXGn Type (%)	XXXG Type (%)	Other ions^c^
**Subfamily Orontioideae**														
*Orontium aquaticum*^d^	49	6	0	39	0	3	0	3	0	0	3	55	45	629 (89%), 659 (13%), 923 (5%)
**Subfamily Lemnoideae**														
*Spirodela polyrhiza*^d^	6	0	0	63	15	3	0	13	0	0	13	6	94	629 (17%), 659 (10%), 689 (16%)
*Landoltia punctata*^d*e*^	7	6	0	25	22	16	11	6	7	0	6	13	87	527 (30%), 689 (15%), 1541 (6%)
*Lemna minor*^d^	40	28	9	11	0	6	0	0	6	0	0	77	23	527 (52%), 629 (38%), 659 (44%)
**Subfamily Pothoideae**														
*Pothos scandens*	12	2	0	75	0	2	0	9	0	0	9	14	86	629 (20%)
*Anthurium* *andraeanum*	20	0	0	74	0	4	0	2	0	0	2	20	80	629 (48%), 923 (6%)
*Anthurium gracile*	31	8	0	46	8	3	0	5	0	0	5	38	62	629 (95%), 923 (14%)
**Subfamily Monsteroideae**							0							
*Rhaphidophora decursiva*	20	1	0	63	0	3	0	13	0	0	13	21	79	629 (45%), 659 (10.%), 923 (5%)
*Scindapsus pictus*	7	5	0	41	0	33	0	2	10	2	4	12	88	629 (4%), 659 (2%)
*Epipremnum aureum*	13	3	0	52	0	20	0	12	0	0	12	16	84	629 (13%), 923 (3%)
*Monstera adansonii*	16	5	0	48	0	26	0	2	2	1	3	21	79	629 (15%), 659 (3%)
**Subfamily Aroideae**							0							
***Zantedeschia *** **CL**														
*Aglaonema costatum*	14	5	0	39	0	33	0	9	0	0	9	19	81	527 (35%)
*Philodendron hederaceum*	13	6	2	49	3	22	0	3	2	0	3	21	79	659 (5%)
*Dieffenbachia seguine*	9	7	0	22	0	48	9	0	5	0	0	16	84	
*Spathicarpa hastifolia*	5	8	0	12	0	43	0	0	32	0	0	13	87	629 (5%), 659 (1%)
***Rheophytes *** **CL**														
*Cryptocoryne aponogetonifolia*^d^	3	3	0	6	0	57	0	0	31	0	0	6	94	629 (2%)
*Lagenandra ovata*^d^	0	3	0	8	0	48	0	0	41	0	0	3	97	659 (2%), 1541 (11%)
***Dracunculus*CL**														
*Amorphophallus dunnii*	3	7	4	11	0	39	2	2	20	12	14	14	86	629 (4%), 659 (1%)
*Syngonium auritum*	7	9	3	29	3	26	0	15	0	8	23	19	81	629 (4%), 821 (1%), 923 (2%)
*Xanthosoma sagittifolium*	9	9	0	24	1	37	0	6	12	2	8	18	82	629 (4%), 659 (1%), 923 (1%)
*Pistia stratiotes*^d^	5	3	0	20	3	28	35	6	0	0	6	8	92	629 (12%), 659 (80%), 689 (9%), 1541 (10%)
*Colocasia esculenta*	13	4	0	41	0	38	0	1	3	0	1	17	83	629 (5%)
*Leucocasia gigantea*	23	0	0	53	0	12	0	12	0	0	12	23	77	
*Alocasia macrorrhizos*	0	10	0	37	0	25	0	18	0	10	28	10	90	
*Pinellia tripartita*	4	5	4	20	0	23	0	8	28	8	16	13	87	629 (4%), 659 (2%)
*Arum pictum*	0	0	0	53	0	13	0	13	0	21	34	0	100	

^a^See Table 1 and Fig. 3

^b^Expressed as a percentage of the total intensities (expressed on a height basis) of ions identified as sodium adduct ions of xyloglucan oligosaccharides

^c^Other ions expressed as a percentage of total ions identified as sodium adduct ions of xyloglucan oligosaccharides: m/z 527 (H_3_), m/z 629 (H_2_P_2_), m/z 659 (H_3_P_1_), m/z 689 (H_4_), m/z 821 (H_4_P_1_), m/z 923 (H_3_P_3_), m/z 1541 (H_6_P_4_); H_x_P_y_, H or Hex = hexose, P or Pent = pentose, x = number of hexoses, y = number of pentoses

^d^Aquatic species

^e^For this species, the XEG and mass spectrometer were those used by Hsieh and Harris (2009, 2012)

### XGs of species in subfamilies Orotioideae and Lemnoideae

Two of the Araceae species in which the XXXG core motif accounted for < 77% were aquatic species in the two earliest emerging subfamilies, Orontioideae and Lemnoideae. These two species had XGs with particularly low percentages of this core motif and were *O. aquaticum* (45% XXXG core motif) in the basal subfamily Orontioideae, and *L. minor* (23% XXXG core motif) in the subfamily Lemnoideae, which is sister to the rest of the Araceae subfamilies (Fig. 3). In the XGO profile of *L. minor*, in addition to XXG and XXGG, XXGGG/XLGG was released with the XXG_n_ core motif. Interestingly however, the XGs in the other two duckweed species examined (subfamily Lemnoideae), *Spirodela polyrhiza* and *Landoltia punctata* had 94% and 87% XXXG core motif, respectively. The xyloglucans of *O. aquaticum*, *S. polyrhiza* and *L. punctata*, but not *L. minor*, were fucosylated, although only XXFG was released, and except for *S. polyrhiza,* in only small proportions. The XGO profiles of the XGs of *L. punctata* and *S. polyrhiza*, but not *L. minor*, were also unusual in having significant percentages of XGOs containing S/D side chains, with *L. punctata* containing XXSG/XXDG (22%) and XLSG/XLDG (11%), and *S. polyrhiza* containing only XXSG/XXDG (15%).

### XGs of species in subfamilies Pothoideae and Monsteroideae

The profiles of the XGs in the species examined of the two subfamilies that form the Bisexual Climbers clade (Fig. 3), Pothoideae and Monsteroideae, both terrestrial, were mostly similar with 79–88% XXXG core motifs and 2–13% fucosylated XGOs, all with XXFG but only two species yielding XLFG. However, the XG of *A. gracile* (subfamily Pothoideae) contained only 62% XXXG core motif and was the third exception in the Araceae species examined to contain < 77%. Another species in the same genus, *Anthurium andraeanum*, had 80%. The XG of *A. gracile* was also unusual in being the only species in the two subfamilies to yield an XGO containing S/D side chains, XXSG/XXDG (8%).

### XGs of species in subfamily Aroideae

Because the most derived subfamily of Araceae, Aroideae, is large, we consider separately the XGO profiles of the XGs of the species in the three major clades, the *Zantedeschia* clade, the Rheophytes clade, and the *Dracunculus* clade.

#### *Zantedeschia* clade

In the *Zantedeschia* clade, the four species examined had 79–87% XXXG core motifs, but two of these *D. seguine* and *S. hastifolia*, both in subclade Spathicarpeae, yielded no fucosylated XGOs and the other two species *Aglaonema costatum* (subclade Aglaonemateae) and *Philodendron hederaceum* (subclade *Philodendron*) yielded XXFG (9 and 3%, respectively), but no XLFG. The xyloglucans of *P. hederaceum* and *D seguine* also yielded XXSG/XXDG (3%) and XLSG/XLDG (9%), respectively.

#### Rheophytes clade

In the Rheophytes clade, both species examined, *C. aponogetonifolia* and *L. ovata* (subclade Cryptocoryneae), are aquatic and their XGs had 94 and 97% XXXG core motifs, respectively, but yielded no fucosylated XGOs and no XGOs with S/D side chains.

#### *Dracunculus* clade

In the *Dracunculus* clade, three of the examined species, *Amorphophallus dunnii*, *Syngonium auritum* and *Xanthosoma sagittifolium*, are in the *Amorphophallus* subclade and the rest in the *Pistia* subclade. Those in the *Amorphophallus* subclade had XGs with 82–86% XXXG core motifs, and all yielded both XXFG and XLFG, with *S. auritum* yielding particularly high total percentages (23%) of both fucosylated XGOs (15% XXFG and 8% XLFG). The XGs of each species in this subclade yielded a small percentage of XGOs with S/D side chains: *A. dunnii* yielded XLSG/XLDG (2%), and *S. auritum* and *X. sagittifolium* yielded XXSG/XXDG (3 and 1%, respectively). The six species examined in the *Pistia* subclade had XGs with 77–100% XXXG core motifs, with *Arum pictum* having 100% and the aquatic species *Pistia stratiotes* having 92%. The XGs of all the examined species yielded fucosylated XGOs, with all but three yielding both XXFG and XLFG. The exceptions yielded only XXFG, but only in small proportions for *P. stratiotes* (6%) and *Colocasia esculenta* (1%). However, this subclade had species with the highest total percentages of both fucosylated XGOs (XXFG and XLFG), with *A. pictum* yielding 34% and *Alocasia macrorrhizos* 28%. The only species in this subclade to yield XGOs with S/D side chains was the aquatic species *P. stratiotes*, which yielded XXSG/XXDG (3%) and a high percentage (35%) of XLSG/XLDG.

### XGs of aquatic species

The XGs of the seven aquatic Araceae species examined all had one or two of the following three unusual features: < 77% XXXG core motif, no F side chains, or > 14% XGOs with S/D side chains. *O. aquaticum* (subfamily Orontioideae) had XG with < 77% XXXG core motif. *L. minor* (subfamily Lemnoideae) had XG with both < 77% XXXG core motif and no F side chains. *S. polyrhiza* and *L. punctata* XG yielded > 14% XGOs with S/D side chains. In subfamily Aroideae, the XG of *P. stratiotes* (clade *Dracunculus*) also had > 14% XGOs with S/D side chains, whereas in the Rheophytes clade *C. aponogetonifolia* and *L. ovata* the XG had no F side chains. However, the lack of F side chains as a feature is not unique to aquatic species as it also occurs in the terrestrial species *D. seguine* and *S. hastifolia*, both in the *Zantedeschia* clade (subclade Spathicarpeae) (see above).

### Other sodium adduct ions

In many of the species examined, sodium adduct ions of other oligosaccharides were found in addition to those of XGOs. Sodium adduct ions [M + Na]^+^ with m/z 527 (Hex_3_), 629 (Hex_2_Pent_2_; XX) (Steck et al. 2021), and 659 (Hex_3_Pent_1_; XGG) were present in substantial proportions in the XGO profiles of some species (Table 2). Smaller proportions of other ions were m/z 689 (Hex_4_), m/z 923 (Hex_3_Pent_3_; XXX or XSG) (Steck et al. 2021), m/z 821 (Hex_4_Pent_1_), and m/z 1541 (Hex_6_Pent_4_). The structural identities of Hex_3_ and Hex_4_ and the source polysaccharide are unknown, but Steck et al. (2021) reported that the *Paenibaccillus* sp. XEG preparation had (1 → 3),(1 → 4)-β-glucanase activity and released oligosaccharides with sodium adduct ions corresponding to Hex_3_ and Hex_4_, probably β-Glc*p*-(1 → 4)-β-Glc*p*-(1 → 3)-Glc*p* and β-Glc*p*-(1 → 4)-β-Glc*p*-(1 → 4)-β-Glc*p*-(1 → 3)-Glc*p*. The XX may be formed from some of the released XXXG oligosaccharides as Steck et al. (2021) found that treatment of XXXG with the *Paenibaccillus* sp. XEG preparation released XX (m/z 629) as well as XG (m/z 494).

## Discussion

The present results show that the XGs of the Araceae are mostly similar in structure to those of the other non-commelinid monocotyledons that have been examined in the orders Asparagales, Liliales, Dioscoreales and Pandanales (Hsieh and Harris 2009). These are fucogalactoxyloglucans with > 76% XXXG core motif and > 23% XXG_n_ core motif. However, our hypothesis that the XGs of all aquatic Araceae species have low proportions (< 77%) of the repeating XXXG core motif [high proportions (> 23%) of the repeating XXG_n_ core motif] and no F side chains, was not supported. The seven aquatic species examined in the present study had XGs that were unusual in having one or two of the following features: a < 77% XXXG core motif, no F side chains, or > 14% XGOs with S/D side chains. *O. aquaticum* in the basal subfamily Orontioideae had XG with < 77% XXXG core motif. The duckweed *L. minor* in the early diverging subfamily Lemnoideae had XG with both a < 77% XXXG core motif and no F side chains. Both *O. aquaticum* and *L. minor* had XGs with particularly low percentages of the XXXG core motif, 45% and 23%, respectively. The two other duckweed species examined *S. polyrhiza* and *L. punctata* had XGs that yielded > 14% XGOs with S/D side chains. In the most derived subfamily Aroideae, where the aquatic habit is a secondary acquisition (Cabrera et al. 2008), the XG of water lettuce *P. stratiotes* (clade *Dracunculus*) also had > 14% XGOs with S/D side chains, whereas in the Rheophytes clade *C. aponogetonifolia* and *L. ovata* the XG had no F side chains. However, XGs with no F side chains were not unique to these species in the Rheophytes clade, *D. seguine* and *S. hastifolia*, both terrestrial species in the *Zantedeschia* clade of subfamily Aroideae, had XGs with no F side chains. S/D side chains also occurred in the XGOs of XGs of a number of other Araceae species, but the percentages of these XGOs did not exceed 14%.

In terms of the phylogeny framework, species having XGs with particularly low percentages of the XXXG core motif occurred in only the basal subfamily Orontiodeae and the early diverging subfamily Lemnoideae. However, *A. gracile*, a terrestrial species in subfamily Pothoideae had XG with < 77% of the XXXG core motif, although another species examined in the same genus, *A. andraeanum*, had XG with > 76% of this motif; the XGs of this genus require further investigation. Other trends in XG structure with phylogeny are less obvious although one should note that the *Dracunculus* clade of the derived subfamily Aroideae had species with XGs that gave particularly high percentages of fucosylated XGOs.

Our results confirmed that the XG of the duckweed *L. minor* had quite a different structure from the XGs of previously examined non-commelinid species (Hsieh and Harris 2009). However, it is interesting that the XGs of the other two duckweed species examined, *L. punctata* and *S. polyrhiza*, had high percentages of the XXXG core motif, XGOs with S/D side chains > 14% and XGOs with F side chains. Further evidence for the fucosylation of *L. punctata* XG was found using immunofluorescence microscopy with the monoclonal antibody CCRC-M1 that specifically recognizes the epitope α-Fuc*p*-(1 → 2)-β-d-Gal*p* present in F side chains (Brennan and Harris 2011; Brennan et al. 2019). As all the species in the duckweed subfamily, Lemnoideae, are floating aquatics, it is unlikely that a single unusual XG structural feature, e.g. a particularly low percentage of the XXXG core motif, is critical for this species to be able to grow in water. Nevertheless, the various unusual XG features may result from an adaptation to the aquatic environment. These plants have high growth rates, mostly clonal reproduction and high rates of DNA substitutions, leading to increased rates of mutation and possibly to novel features in cell wall polysaccharides (Nauheimer et al. 2012; Avci et al. 2018).

Interestingly, the structures of another family of polysaccharides in primary cell walls, the pectic polysaccharides, have also been shown to vary within the Lemnoideae (Avci et al. 2018). This study was carried out in the context of the phylogeny of this subfamily (Tippery et al. 2015) and it was found that *L. minor*, *L. punctata*, and *S. polyrhiza* all contained high proportions of the unusual pectic polysaccharide apiogalacturonan, whereas species of the more derived genus *Wolfiella* and the most derived genus *Wolffia* have much lower proportions of apiogalacturonan, with *Wolffiella* having pectic polysaccharides with a high content of arabinosyl residues that are likely from arabinan side chains of rhamnogalacturonan I and *Wolffia* having high proportions of xylogalacturonan. As we have argued above for the unusual XG in *L. minor*, Avci et al. (2018) have argued that because *Wolffia* spp, *L. minor*, *L. punctata*, and *S. polyrhiza* can all occur in the same pond, apiogalacturonan is not critical for the growth of these plants in water.

In addition to the Araceae subfamily Lemnoideae, apiogalacturonans have been found in the pectic polysaccharides of other plants in the order Alismatales. These are the seagrasses, five families of marine plants (Cymodoceaceae, Hydrocharitaceae, Posidoniaceae, Ruppiaceae and Zosteraceae) (Pfeifer and Classen 2020; Pfeifer et al. 2022). However, little is known about the structures of the XGs in these plants, but immunofluorescence microscopy with the monoclonal antibodies LM15 that specifically recognizes XG and CCRC-M1 that specifically recognizes F side chains (see above) showed that in the leaves of *Zostera muelleri* (Zosteraceae), although all primary walls contained XGs only those of the phloem cells had F side chains (Brennan and Harris 2011; Brennan et al. 2019).

In the results of the present study, we refer to S/D side chains in XGOs because it is not known if the arabinose (Ara) residues are in the furanose (Ara*f*) form (side chain S) or in the pyranose (Ara*p*) form (side chain D). As far as we are aware, S side chains have not previously been reported in XGs of monocotyledons, but they have been found in XGs of species in the eudicotyledon orders Solanales, Lamiales, and Gentianales (Hoffman et al. 2005), which are all in the Lamiids subclade of the Asterids clade (APG IV 2016). The S side chains have been found in XGs with a repeating XXXG core motif in *Olea europaea* (olive) (family Oleaceae) in the Lamiales (Vierhuis et al. 2001) and in *Nerium oleander* (family Apocynaceae) in the Gentianales (Hoffman et al. 2005), with the XGOs XXSG and XLSG being released. Because S, but not D side chains have been found in these angiosperms, it is likely that S rather than D side chains occur in the Araceae XGs. Outside the angiosperms, S side chains have been identified in the XGs of the monilophyte (ferns *sensu lato*) *Ceratopteris richardii*, where only the gametophyte generation was examined, and *Equisetum hyemale*, where it occurred in only small proportions (Peña et al. 2008). However, either S or D side chains have also been reported in the XGs of two other monilophytes *Azolla filiculoides* and *Microsorum punctatum* (Hsieh and Harris 2012). D side chains have not previously been reported in the XGs of any seed plants, but they have been identified in the XGs of some lycophyte species and *E. hyemale* (Peña et al. 2008).

The biological functional significance of the different repeating core motifs and side chains in XGs is unknown, because with one exception, the functions of XGs are uncertain. The exception is the function of the XGs that occur in the thick cell walls of cotyledons in the seeds of many eudicotyledons. These XGs are hydrolysed during germination and serve as a reserve carbohydrate. Nevertheless, for many years, the XGs in primary cell walls of vegetative organs were considered to bind to cellulose microfibrils and form tethers between adjacent microfibrils, which control cell wall expansion during diffuse growth (Fry 1989; McCann et al. 1990). However, there is now evidence that does not support this function of XGs (Park and Cosgrove 2015; Zhang et al. 2021; Cosgrove 2022). In particular, mutants of *Arabidopsis thaliana* containing no detectable XG in their cell walls were morphologically very similar to the wild types (Cavalier et al. 2008; Kim et al. 2020). Consistent with this, recent coarse-grained molecular dynamics simulations of cell walls indicate that tensile forces are transmitted mostly by direct contacts between cellulose microfibrils rather than by XG tethers (Zhang et al. 2021).

In conclusion, our hypothesis was not supported. However, all of the aquatic species examined had XGs that were unusual in having either one or two of the following features > 23% of the repeating XXG_n_ core motif, no F side chains, or > 14% XGOs with S or D side chains. Only the XG of *L. minor* had two of these features: > 23% XXG_n_ core motif and no F side chains. Furthermore, XGs with no F side chains were found not to be a character unique to aquatic Araceae species. Particularly high proportions of the XXG_n_ core motif were found in the XGs of two aquatic species, *O. aquaticum* (55%) in the basal subfamily Orontioideae and of *L. minor* (77%) in the early emerging subfamily Lemnoideae. Such high proportions may occur only in the XGs of species in these subfamilies, whereas the XGs of the other two species examined in the Lemnoideae, *S. polyrhiza* and *L. punctata* had low proportions of the XXG_n_ core motif, 6% and 13%, respectively. High proportions (> 14%) of XGOs with S or D side chains were also released from the XGs of *S. polyrhiza* and *L. punctata*, as well as from the XG of *P. stratiotes* in the derived subfamily Aroideae. Thus, there appears to be no clear-cut phylogenic trend in the XG structures of aquatic species in this family.

### *Author contribution statement*

SYH, JL, DW and PHL performed the experiment. SYH, JI, BI, HCL, MK, CHC, PHL, PJH and YH analysed the data. SYS, PJH and YH wrote the manuscript.

## Supplementary Information

Below is the link to the electronic supplementary material.

Supplementary file1 (DOCX 1487 KB)

## Data Availability

The authors declare that all data supporting the findings of this study are available within the article and its supplementary information.

## Abbreviations

AIR: Alcohol-insoluble residue
XEG: Xyloglucan-specific *endo*-(1 → 4)-β-glucanase
XG: Xyloglucan
XGO: Xyloglucan oligosaccharide
