# Supplementary material for: Structures of the xyloglucans in the monocotyledon family Araceae (aroids)
Source: Planta. 2023 Jan 17;257(2):39. doi: 10.1007/s00425-023-04071-w (PMC9845173; doi:10.1007/s00425-023-04071-w)
Supplement: Supplementary file 1 — Supplementary file1 (DOCX 1487 KB) [file 425_2023_4071_MOESM1_ESM.docx]

**Supplementary Material**

**Structures of the xyloglucans in the monocotyledon family Araceae (aroids)**

Shih-Yi Hsiung^a,b^, Jing Li^a,c^, Balazs Imre^a,b^, Mu-Rong Kao^a,b^, Hsien-Chun Liao^d^, Damao Wang^e^, Chih-Hui Chen^d^, Pi-Hui Liang^f^, Philip J. Harris^g^, Yves S. Y. Hsieh^* a,b^

^a^ Division of Glycoscience, Department of Chemistry, School of Engineering Sciences in Chemistry, Biotechnology and Health, KTH Royal Institute of Technology, AlbaNova University Centre, Stockholm, SE106 91, Sweden

^b^ School of Pharmacy, College of Pharmacy, Taipei Medical University, Taipei, Taiwan

^c^ College of Life Science, Shanghai Normal University, Shanghai, China

^d^ Division of Botany, Taiwan Endemic Species Research Institute, Nantou 552, Taiwan

^e^ College of Food Science, Southwest University, Chongqing, China

^f^ School of Pharmacy, College of Medicine, National Taiwan University, Taipei, Taiwan

^g^ School of Biological Sciences, The University of Auckland, Auckland Mail Centre, Private Bag 92019, Auckland 1142, New Zealand

^*^Author for correspondence Yves S.Y. Hsieh: Email yvhsieh@kth.se


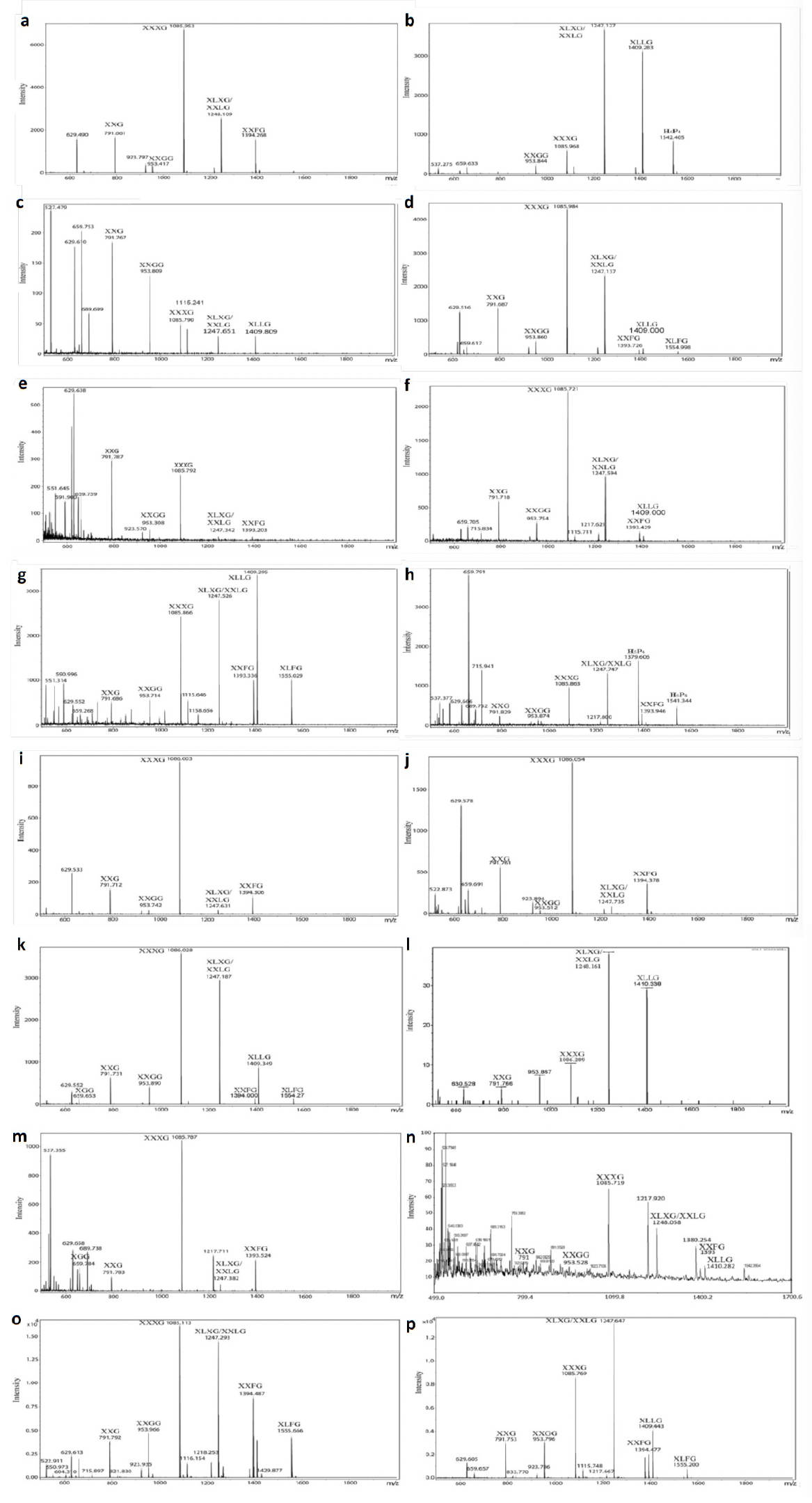
**Fig. S1**

**Fig. S1** MALDI-TOF mass spectra of xyloglucan oligosaccharides obtained from AIRs, from the other 16 species examined, treated with XEG (genera arranged in alphabetical order); HxPy = Hexose x and Pentose y). **a** *Epipremnum aureum* (subfamily Monsteroideae). **b** *Lagenandra ovata* (subfamily Aroideae). **c** *Lemna minor* (subfamily Lemnoideae). **d** *Monstera adansonii* (subfamily Monsteroideae). **e** *Orontium aquaticum* (subfamily Orontioideae). **f** *Philodendron hederaceum* (subfamily Aroideae). **g** *Pinellia tripartita* (subfamily Aroideae). **h** *Pistia stratiotes* (subfamily Aroideae). **i** *Pothos scandens* (subfamily Pothoideae). **j** *Rhaphidophora decursiva* (subfamily Monsteroideae). **k** *Scindapsus pictus* (subfamily Monsteroideae). **l** *Spathicarpa hastifolia* (subfamily Aroideae). **m** *Spirodela polyrhiza* (subfamily Lemnoideae). **n** *Landoltia punctata* (subfamily Lemnoideae). **o** *Syngonium auritum* (subfamily Aroideae). **p** *Xanthosoma sagittifolium* (subfamily Aroideae).
